# Frequency-Invariant Representation of Interaural Time Differences in Mammals

**DOI:** 10.1371/journal.pcbi.1002013

**Published:** 2011-03-17

**Authors:** Hannes Lüling, Ida Siveke, Benedikt Grothe, Christian Leibold

**Affiliations:** 1 Department of Biology II, Ludwig-Maximilians Universität München, Planegg-Martinsried, Germany; 2 Bernstein Center for Computational Neuroscience Munich, Planegg-Martinsried, Germany; University College London, United Kingdom

## Abstract

Interaural time differences (ITDs) are the major cue for localizing low-frequency sounds. The activity of neuronal populations in the brainstem encodes ITDs with an exquisite temporal acuity of about 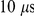. The response of single neurons, however, also changes with other stimulus properties like the spectral composition of sound. The influence of stimulus frequency is very different across neurons and thus it is unclear how ITDs are encoded independently of stimulus frequency by populations of neurons. Here we fitted a statistical model to single-cell rate responses of the dorsal nucleus of the lateral lemniscus. The model was used to evaluate the impact of single-cell response characteristics on the frequency-invariant mutual information between rate response and ITD. We found a rough correspondence between the measured cell characteristics and those predicted by computing mutual information. Furthermore, we studied two readout mechanisms, a linear classifier and a two-channel rate difference decoder. The latter turned out to be better suited to decode the population patterns obtained from the fitted model.

## Author Summary

Neuronal codes are usually studied by estimating how much information the brain activity carries about the stimulus. On a single cell level, the relevant features of neuronal activity such as the firing rate or spike timing are readily available. On a population level, where many neurons together encode a stimulus property, finding the most appropriate activity features is less obvious, particularly because the neurons respond with a huge cell-to-cell variability. Here, using the example of the neuronal representation of interaural time differences, we show that the quality of the population code strongly depends on the assumption — or the model — of the population readout. We argue that invariances are useful constraints to identify “good” population codes. Based on these ideas, we suggest that the representation of interaural time differences serves a two-channel code in which the difference between the summed activities of the neurons in the two hemispheres exhibits an invariant and linear dependence on interaural time difference.

## Introduction

The neuronal representation of the azimuthal position of a low-frequency sound source has been extensively studied across many mammalian and avian species [1], [2], [3], [4], [5], [6], [7], [8], [9]. There is general agreement that the stimulus parameter that carries most of this positional information is the interaural time difference (ITD), which is produced by the disparity of travelling times from the sound source to the two ears [10], [11], [12]. It is also unquestioned that ITDs are neuronally represented via a firing rate pattern in populations of neurons in the brainstem. In mammals the underlying binaural coincidence detection takes place in the superior olivary complex both in the medial superior olive (MSO) [1], [3], [7] and the low-frequency regions of the lateral superior olive [13]. In birds the binaural coincidence detection is performed in the Nucleus laminaris [4], [8], which is analogous to the MSO [14], [15]. The way that ITDs are exactly represented by the firing rates of neuron populations in the brainstem is still a matter of debate and is presumed to vary across species [16], [17], [18]. Presently all quantitative coding theories have only considered ITD representations for stimuli with fixed spectral content [18], [19], [20]. Those theories showed that the psychophysical acuity can be explained by the rate statistics of the best single neurons. The firing rates of ITD encoding neurons are, however, strongly altered by changes in stimulus frequency [2] as well as many other factors such as sound level [1], [21], interaural level difference [22] and the presence and type of concurrent sounds [23], [24]. Taking into account additional stimulus dimensions complicates coding theories, because different activity patterns encode for the same ITD and thus the one-to-one relation between the firing rate of a single neuron and the stimulus ITD is lost.

Here we develop a theory of ITD representation that is invariant to one additional stimulus dimension: the frequency of a pure tone. We compare encoding on the single-cell level with two population encoding schemes. We find that single-cell mutual information only roughly accounts for the observed variability of the response properties. The population patterns, however, are consistent with the idea of a two-channel code, in which the stimulus ITD is linearly represented by the difference of the summed activities in each hemisphere.

## Results

The following analyses are based on recordings from the dorsal nucleus of the lateral lemniscus (DNLL) of Mongolian gerbils (Meriones unguiculatus). The DNLL is one stage downstream to the superior olivary complex and receives input from both the MSO and the lateral superior olive. Binaural DNLL responses have been shown to reflect the ITD sensitivities of superior olivary complex neurons well [25]. The data was obtained from 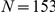 single neurons from 41 animals (see Materials and Methods). In brief, we used pure tone stimuli with frequencies covering 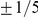 of an octave around the neuron's best frequency (BF). Stimuli were delivered binaurally at an interaural intensity difference of 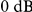 and varying ITDs.

**Figure 1:**
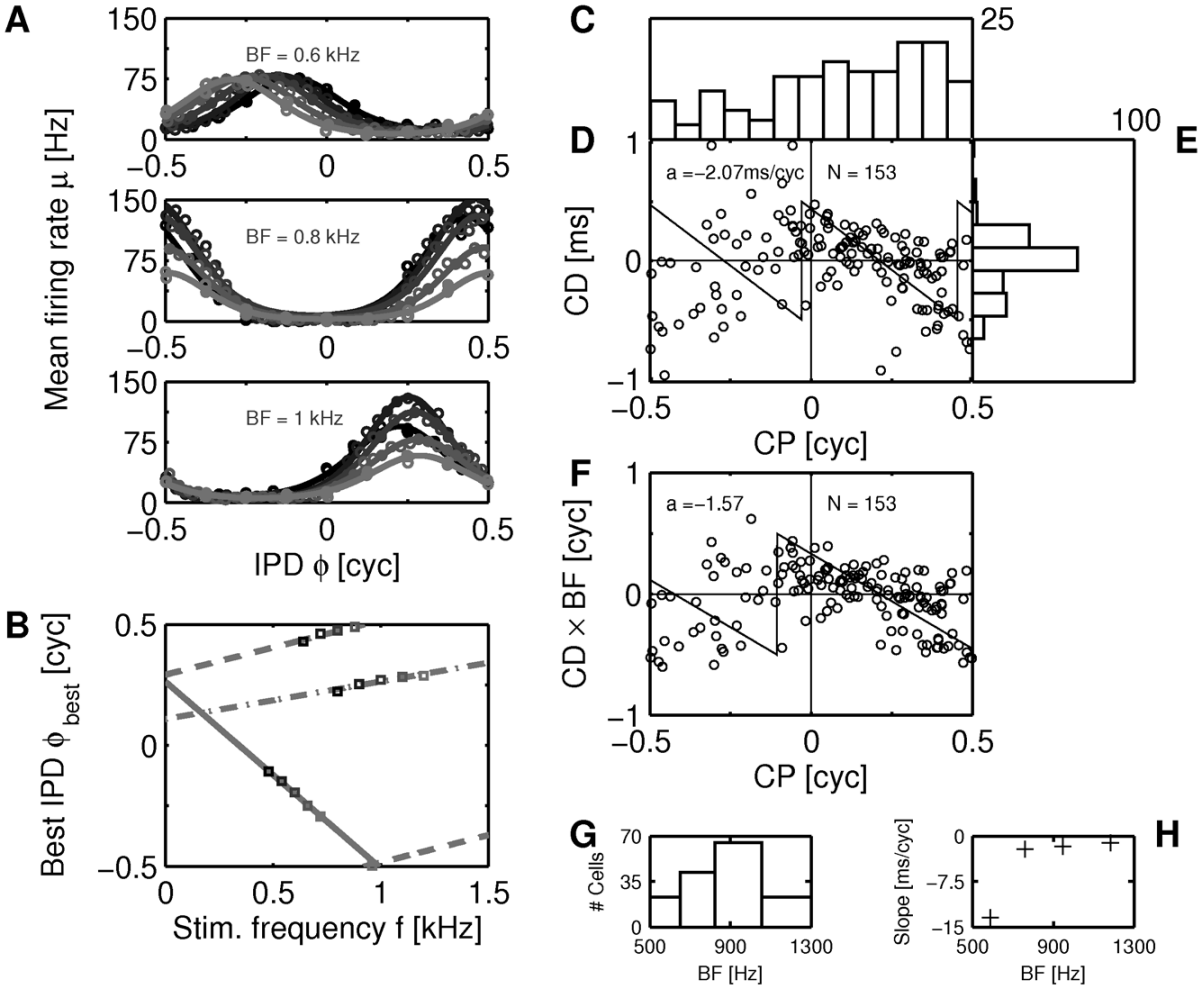
Frequency-dependence of ITD sensitivity. (A) Tone delay functions for three exemplary DNLL neurons using five stimulus frequencies (dark to light grey indicates low to high frequency) centered at BF. Circles depict the means of measurements, the solid lines show a cyclic Gaussian fit (see Materials and Methods). (B) Best IPD vs. stimulus frequency (phase-frequency curves) for the three neurons from A (corresponding to the three different line styles). Note that the phase axis is cyclic. (C) Distribution of characteristic phases (CPs) for 153 DNLL neurons. (D) CPs and characteristic delays (CDs) exhibit circular-linear correlation. Best circular-linear fit is depicted by the solid line (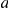: slope). (E) Distribution of CDs. (F) Correlation between CP and 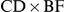. (G) Histogram of BFs. (H) Average slopes 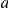 of the CP, 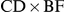 distribution in the four frequency bands from G.

The sensitivity of a single neuron to the ITD 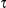 of the pure tone stimulus with frequency 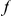 is shown by the tone delay function, which measures the neuron's discharge rate as a function of the applied interaural phase difference (IPD) 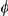 which is the product of ITD and stimulus frequency 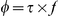. Tone delay functions in the brainstem strongly depend on the frequency of the stimulus (Figure. 1A). Classically, this frequency dependence of the tone delay functions is quantified via the best IPD 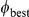 at which the tone delay function of a neuron takes its maximum. In the superior olivary complex and the DNLL, the best IPD of single neurons changes approximately linearly with frequency 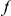 ([3], [13], [25], [26], [27], [28] and Figure 1B),
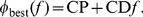
(1)


The parameters describing this linear relationship are the characteristic phase (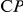) and characteristic delay (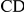). They are obtained by circular-linear regression between the circular variable 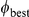 and the linear variable 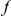 (see Materials and Methods). For a pure delay line model as suggested by Jeffress [29] CD would be the difference of transmission delays from the two ears to a coincidence detector neuron, whereas CP should be zero. On the other hand, cells that receive inhibition from the contralateral ear and excitation from the ipsilateral ear would exhibit CP = 0.5. The distribution of CPs and CDs from our own data are quite different from these theoretical predictions. The CPs are distributed over the whole cycle with a bias towards positive phases (Figure 1C). The distribution of CDs peaks at zero and is skewed to negative CDs (Figure 1E). A circular-linear fit revealed a negative correlation between CDs and CPs (Figure 1D) with a slope of about 2 ms per cycle (mean resultant length 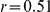, linear-circular correlation 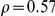, 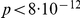 [30]).

A slightly larger correlation (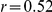) was found between CP and the product 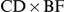 (Figure 1F), which suggests a tonotopy in characteristic delays (as also reported in [31]). A correlation between BF and CD was further corroborated by splitting up the data into four frequency bands with a width of 1/4 of an octave (Figure 1G) and computing the circular linear fits in each of these bands. The slopes of these fits correlate (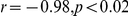) with the mean BF in the bands suggesting that large CDs predominantly occur in low-frequency bands (Figure 1H). For the further analysis we therefore considered the frequency-scaled parameter 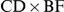 instead of CD.

### Single-cell mutual information

To understand how the observed distribution of CPs and CDs affects the encoding of ITDs, we calculated the mutual information between stimulus ITD 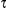 and the corresponding firing rate 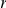 of a single cell,
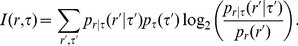
(2)


**Figure 2:**
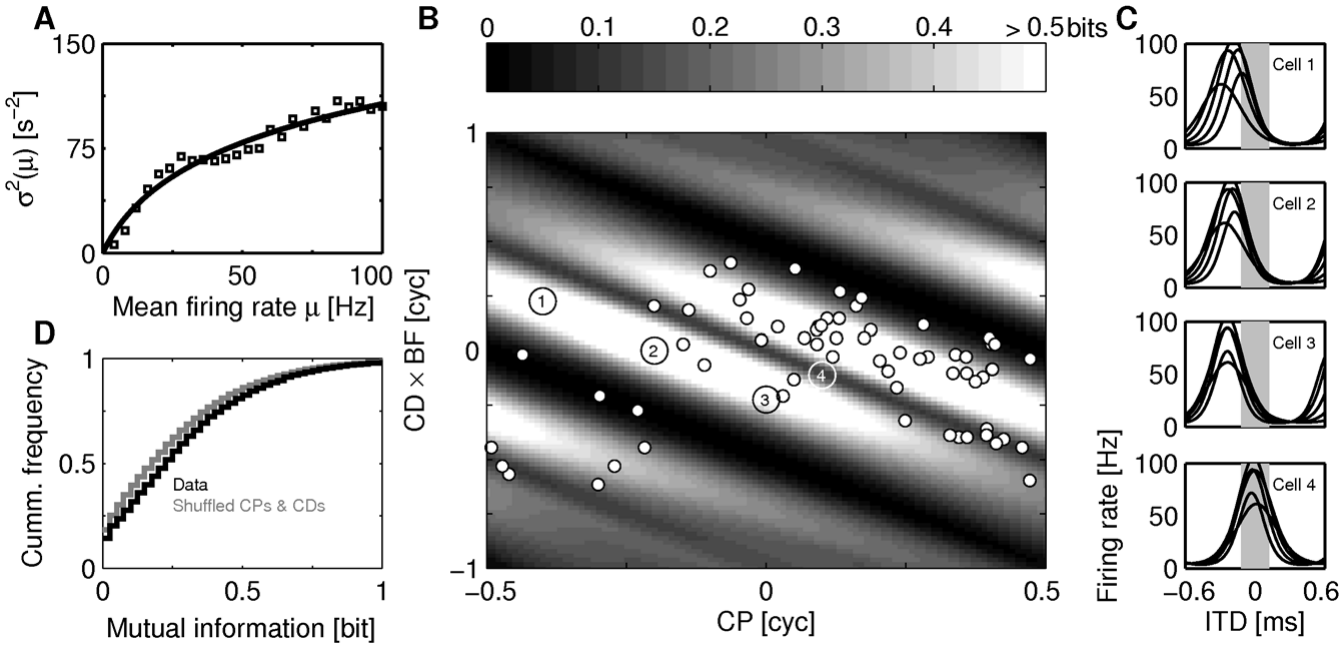
Rate statistics and mutual information. (A) Variance 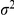 of the rate distributions as a function of mean firing rate 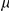 (squares averaged over all 153 neurons) and a logarithmic fit (solid line). For low rates the slope of the variance is consistent with a Poisson process (see Materials and Methods). (B) Mutual information (MI; grey levels) as a function of CP and 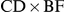 for average fit parameters of the tone delay function in the best frequency band between 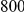 and 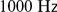. Circles illustrate the distribution obtained from the DNLL population in this best frequency band. (C) Firing rate as a function of ITD for four exemplary combinations of CP and CD (large circles with numbers in B). Grey bars indicate the physiological range of ITDs. (D) Cumulative distributions of mutual information of the real CP-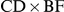 pairs (black) and for 1000 repetitive shuffles of CP and 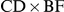 (grey).

The prior distribution 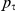 of ITDs was obtained by assuming uniformly distributed dihedral angles (see Materials and Methods). The prior depends on the inter-ear distance 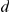 that determines the maximal accessible ITD 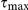 and thus the physiological range of ITDs 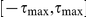. We constructed the conditional distribution 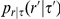 of observing a rate 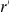 at a given ITD 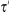 from the recorded firing rates as follows. We first fitted the mean tone delay functions 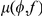 by a cyclic Gaussian (Figure 1A). We then pooled all recording conditions (ITD and stimulus frequency) that led to the same mean firing rate 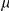 in one neuron and constructed neuron-specific Gaussian rate distributions 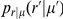 by fitting the variance of the rate (Figure 2A and Materials and Methods). From 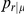 we constructed conditional distributions 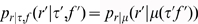 of firing rates 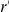 for given ITD 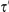 and stimulus frequency 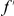. The distributions 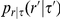 were obtained by averaging over frequency, which reflects the assumption that input frequencies are distributed uniformly under natural stimulus conditions (the differences for 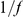 distributed frequencies are only minor; see Supporting Information Figure S1). All our analyzes were done for neurons in the (best) frequency band between 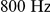 and 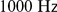 in which we had the most cells (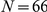). This distinction between frequency bands was necessary since the shape of the tone delay function in the physiological phase range strongly depended on the BF of the neuron. Nevertheless, the distributions for the other frequency bands was qualitatively similar as far as we can tell from the limited sample sizes (see Supporting Information Figure S1).

Figure 2B illustrates the mutual information for arbitrary pairs of CP and CD using a phase delay function 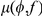 with the average fit parameters of the population of neurons. Since the formalism is symmetric with respect to both hemispheres, the mutual information plot is mirror symmetric in the CP-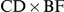 plane. The bright regions with high frequency-invariant information show distinctly negative slopes. The steepness of these slopes is roughly 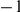, i.e. 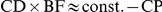. All neurons along such a line thus have the same best phase
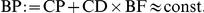



In the case of Figure 2B, this constant best phase equals about 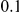 cycles.

To understand what gives rise to high mutual information in these regions, we plotted examples for cells with high and low mutual information (Figure 2C). The “synthetic” cells with high mutual information have the steepest slopes of their rate response in the physiological range. The response functions of the cell with highest mutual information (cell 2) are very similar for all frequencies in the physiological range, which is indicative for the frequency invariance of the ITD representation for this single neuron. In general, however, the response of single neurons is not frequency invariant, even for those with high mutual information. The example cell in Figure 2C with low mutual information (cell 4) exhibits the peak in the physiological range.

Figure 2B also shows that the values of CP and 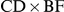 are not concentrated around the position of largest mutual information, nor do they follow exactly the line with slope -1. Some of the cells are even located at regions of the CP-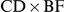 plane with very low mutual information. We therefore quantified how much, if at all, the experimentally observed distribution of CPs and CDs provides an advantage for a frequency-invariant decoding of ITDs in terms of single-cell mutual information. We generated mutual information values for a surrogate set of 1000 cells, which was obtained by shuffling the fit parameters of the mean tuning curves 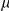, while keeping CP and 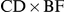 constant. The mutual information of this control set was compared to that of a second surrogate set with shuffled CPs and CDs (Figure 2D). The gain in single-cell information due to the observed CP-CD distribution is rather small (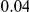 bits on average) but significant (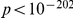, Kolmogorov-Smirnoff test). We then set all CPs to zero without changing the CDs, which would correspond to an idealized Jeffress-like situation with only delay lines and no additional phases. For such a setting, we find that the mean mutual information would also become slightly worse by 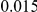 bits (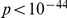, Kolmogorov-Smirnoff test) as compared to the measured distribution.

**Figure 3:**
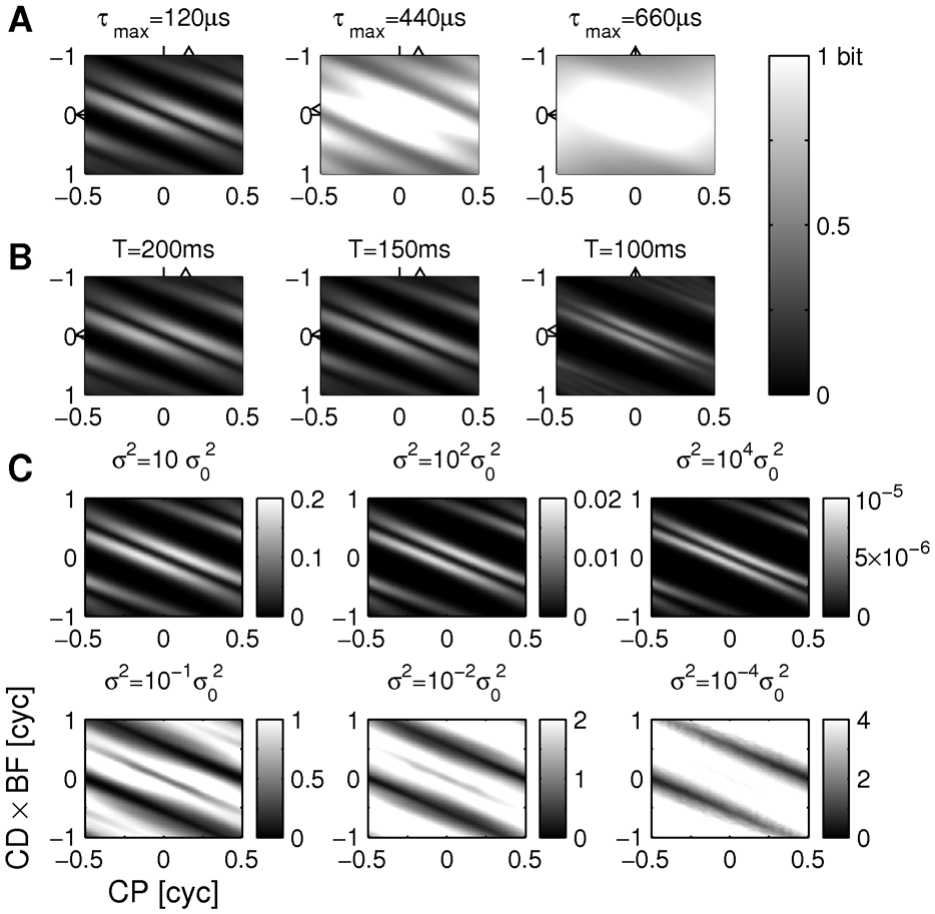
Parameter dependence of mutual information (MI). (A) MI as a function of CP and 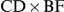 for three different inter ear distances and the respective physiological ranges 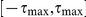. The left plot is a copy of Figure 2D. Axis are the same for all subplots (see bottom left of C). The triangles on the top indicate the CP of maximum mutual information, the ticks on the top indicate CP = 0. Triangles and ticks at the vertical axis indicate the analogous CD values. (B) MI for three different stimulus lengths. Again, the left plot corresponds to the default case from Figure 2D. Triangles follow the same convention as in A. The best phases (BP) are 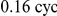 (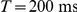), 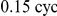 (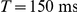), and 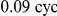 (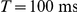). (C) MI for six different noise levels. Noise is defined as multiple of the variance 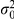 of the default case from Figure 2D. Note that the MI is depicted with different grey scales (in bits).

Next, we wanted to understand how much our single cell results are determined by the type of animal and our stimulus. We thus studied how the single-cell mutual information depends on inter-ear distance 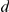 and stimulus length 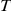. At first we evaluated the influence of 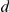. From equation (2), we know that the inter-ear distance 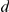 influences the mutual information via the prior distribution 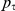 of ITDs. We used the firing statistics 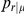 determined from the gerbil DNLL recordings to make predictions about the population pattern of mutual information for hypothetical animals with larger inter-ear distances than that of the gerbil. The largest considered value gave rise to a maximal ITD 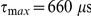, which roughly corresponds to the situation in humans. We find the general tendency that for larger 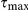 the stripe-like organization becomes less pronounced and the two clusters of high mutual information fuse into one (Figure 3A). This fusion is associated with the maximum of the mutual information moving towards smaller characteristic phases. We thus conclude that CPs different from zero are particularly useful for animals with small head size. For animals with larger heads, cells with large non-zero CPs still convey ITD information. However, they are less essential, since ITD information is also available for small CPs.

In a second analysis, we made predictions for reduced stimulus length in that we only considered the activity recorded during the initial interval of length 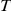 (Figure 3B). The stripe-like pattern in the mutual information profiles is present for every considered interval length 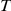. With decreasing interval length, we find a reduction in both the separation of the stripes with high mutual information and their thickness. In other words, a decrease in 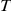 makes the maximum mutual information move towards smaller CPs and the peaks of the phase delay function move into the physiological range.

To find out whether the changes induced by the reduction of 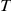 are due to different tone delay functions for onset and sustained firing, or mainly attributable to the increase of noise, we also calculated mutual information for scaled noise levels (Figure 3C). For artificially increased noise levels the results were similar to those in Figure 3B for decreasing duration 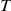; both separation and thickness of the stripes with high mutual information are reduced. However, for artificially decreased noise levels, the stripes with high mutual information not only become thicker and fuse together but also the maximum mutual information moves towards larger characteristic phases. As a consequence, regions with high mutual information also occur for both large positive CP and CD and very negative CP and CD (see Discussion).

To summarize, single cells in the DNLL do generally not exhibit frequency invariant ITD representations. However, the single cell mutual information suggests that the observed distribution of CPs and CDs is particularly suited to conserve frequency-invariant ITD information. We thus propose that a frequency invariant ITD representations may be found by testing appropriate readout models for the population of DNLL responses.

### Population codes

The single cell analysis has revealed two obvious problems: 1) Some of the cells have very low values of mutual information. 2) It is unclear why not all of the cells cluster at the CP-CD position with maximal mutual information. To address these concerns we next studied the coding capabilities of DNLL neurons on the level of a population using simulated firing rate patterns of 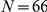 neurons in the frequency band between 800 and 1000 Hz (Figure 1G) for different ITDs and stimulus frequencies.

**Figure 4:**
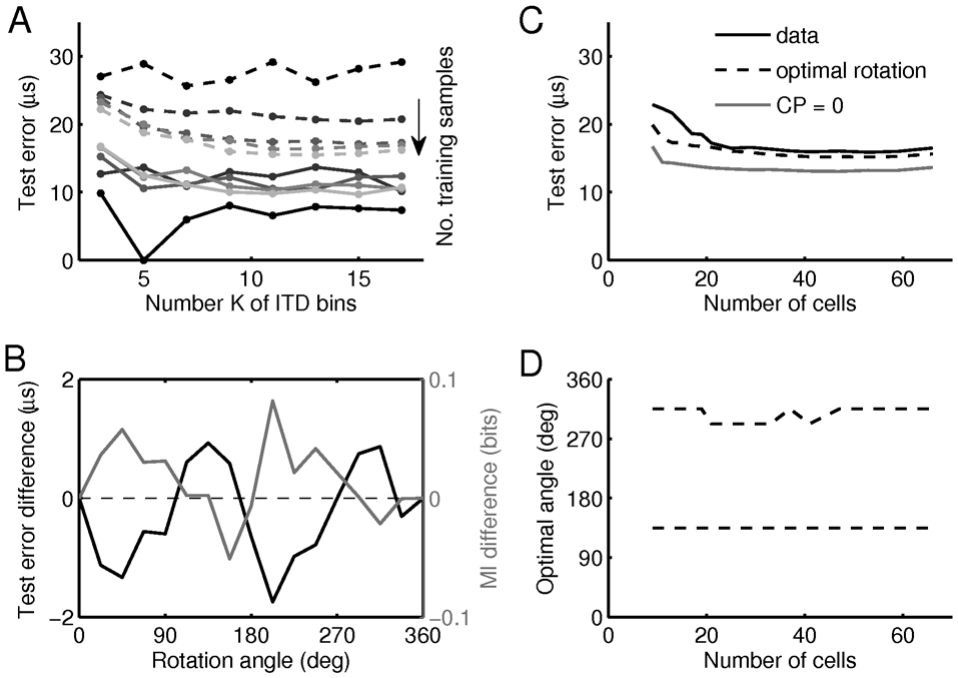
Linear separability of population patterns. (A) Test error as a function of the number 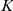 of classifications bins for five different numbers of training samples (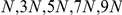 as indicated by grey level). Training errors are plotted as solid lines, test errors (localization acuity) are plotted as dashed lines. (B) Difference in test errors (black) and single-cell mutual information (grey) as a function of the rotation angle. Positive differences indicate that the value obtained with the non-rotated CP-CD distribution is larger. For the linear classifiers, the number of training samples was 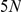. (C) Test error as a function of the number of input neurons for the actually measured CP-CD distribution (solid line), for the optimal rotation angle (dashed line), and for CP set to zero (grey). (D) Optimal rotation angles at which the test error difference from B has local maximum.

First we used the rate patterns as input vectors to linear classifiers (Figure 4A) that then produced a labelled line code with “grandmother neurons” that encode one specific azimuthal position. For this purpose, linear support vector machines [32], [33] were trained in a one-vs.-one paradigm to classify the population patterns according to their underlying ITD into 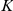 categories (azimuth bins). The ITD resolution 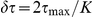 of the labelling scheme therefore is inversely proportional to the number of categories. As expected, the test error (predicted acuity) decreased and the training error increased with the number of training samples both saturating at a number of about 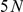 (Figure 4A). The test error converges to an acuity of about 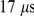, which is in agreement with the psychophysical acuity of gerbils of about 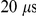 [34], [35]. This final acuity is reached at roughly 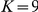 ITD bins.

To show how much the negatively correlated distribution in the CP-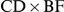 plane contributes to the test error we again shuffled the CP values, however, we did not find a significant change of the test error. We therefore carried out further manipulations in the CP-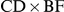 space. First, we set all CPs to zero mimicking the distribution of the idealized Jeffress model. Surprisingly, this manipulation accounted for an improvement of about 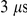 of (root-mean-square) acuity (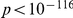, t-test for 150 repeats). The Jeffress model would thus be better suited than the actually observed DNLL population patterns if neurons in higher-order nuclei acted as linear classifiers, or equivalently, if higher-order centers exhibited grand mother cells that fire specifically for small ITD intervals.

Second, we monitored the change of test error for rotated CP-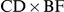 distributions that were constructed by rotating the CP-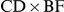 position vectors of all single neurons by the same angle (Figure 4B). For rotation angles about 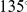 and 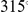 the manipulated CP-CD distribution gave rise to about 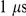 improved acuity as compared to the unrotated case. These optimal angles also roughly coincide with the rotation angles for which we also find the mean single-cell mutual information to be maximal. For none of the rotation angles, however, is the acuity as good as for the Jeffress-type scheme with CP = 0.

A possible explanation for the above non-optimality of the population rate code is that a faithful frequency-invariant decoding of ITD could require less than the 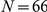 neurons that we have used as input to our linear classifier, i.e. the observed CP and CD values could be optimal for smaller subpopulations. We therefore retrained the classifiers with fewer input neurons. Figure 4C depicts the mean acuity of the linear classifier as a function of the number of input units for both the actual and the optimally rotated CP-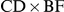 distributions. For each number of input neurons, we chose the subset with highest values of single-cell mutual information. The acuity decreases with subset size but quickly saturates at about 25 neurons. There it is only slightly worse (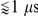) than the optimally rotated CP-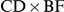 distribution. The optimal rotation angles are independent of the subset size (Figure 4D). The acuity for the Jeffress case (CP = 0), however, is always about 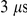 better.

Interestingly, we also do not observe a correlation between single cell mutual information and the weights of the classifiers (not shown). This means that only very few features of the population representation seem to be sufficient for the classifier to detect the right ITD and the classifiers may learn different features of the population pattern for each frequency.

From the above findings we conclude that 1) the actually observed distribution of CPs and CDs is not optimal in terms of the readout acuity of linear classifiers 2) only a small subset of cells would be sufficient to achieve best acuity.

**Figure 5:**
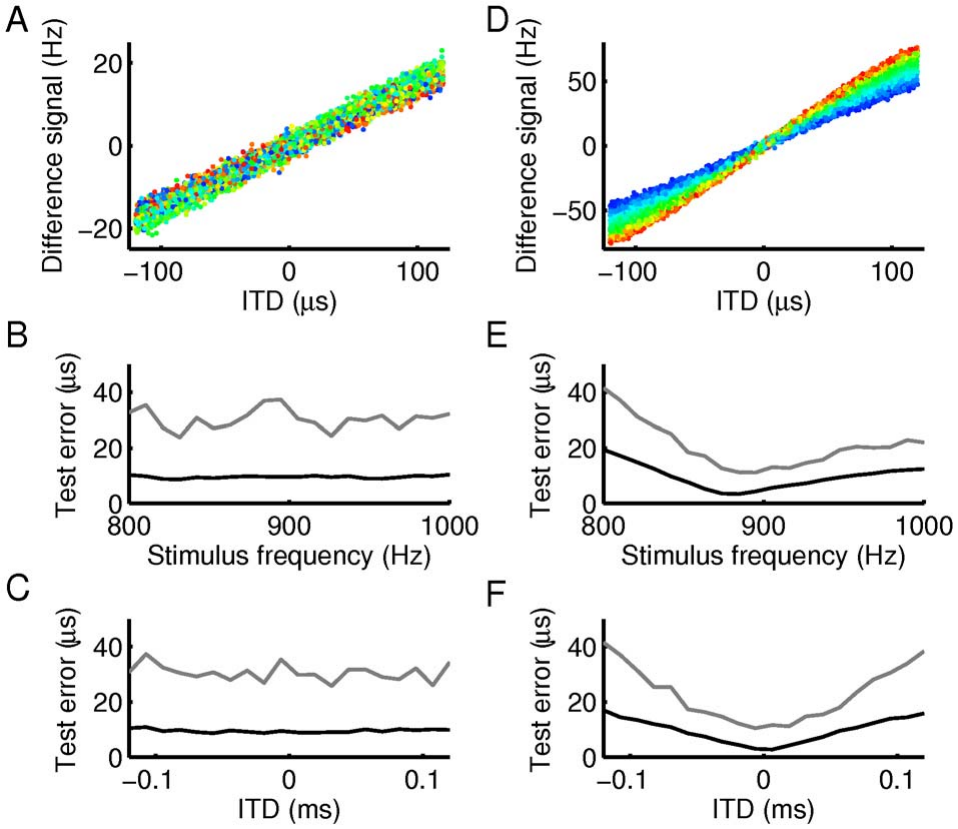
Bilateral difference coding. (A) Difference between the mean rate of the contralateral and the ipsilateral DNLL population. Colors indicate stimulus frequency from 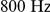 (blue) to 1000 Hz (red). (B) Test error as a function of frequency (black: root-mean-square error, grey: maximal error). (C) Test error as a function of of ITD (black: root-mean-square error, grey: maximal error). (D–F) Same as A-C where all neurons are simulated using the combination of CP and CD with highest mutual information.

As a second way to interpret the population rate pattern we considered a bilateral difference model (or two-channel model) [35], [36], in which the total activity in one brain hemisphere is subtracted from that in the other hemisphere. Again, we concentrated on the frequency band between 800 and 1000 Hz, since there the distribution of best frequencies was pretty much flat and does not induce a sampling bias. We again simulated firing rate patterns for different ITDs and stimulus frequencies based on the rate distributions of the DNLL neurons. The bilateral (rate) difference signal 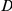 was computed as the mean firing rate in the population of simulated neurons minus the mean rate for an identical population in the opposite hemisphere (with mirrored CP and CD). The relation between the stimulus ITD 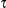 and the difference signal 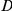 is very well represented by a linear function (Figure 5A). The least squares fit 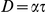 thus provides a linear estimate of the stimulus ITD 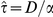. The test error between 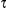 and its estimate 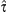 is largely independent of the stimulus frequency and ITD (Figure 5B, C).

We next recomputed the test error for a hypothetical population with all neurons having the same optimal combination of CP and CD at which the mean mutual information from Figure 2D is maximal. The relation between difference signal and ITD is no longer linear and clearly depends on the stimulus frequency (Figure 5D). As a result also the test error depends non-monotonically on frequency with a minimum at the center frequency of the band (Figure 5E). The observed variability in CP and CD thus is responsible for the frequency-invariant linear relation between ITD and the difference signal. Moreover, this linearity is robust with respect to a small jitter in the CD and CP values (Supporting Information Figures S2 and S3) and, hence, this property does not depend on the exact distributions measured. Figure S2 (Supporting Information) also shows that a similar linear difference signal is obtained from the smaller subset (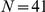) of units measured with best frequencies between 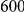 and 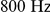 indicating that the linear readout is not a specialty of the considered best frequency band.

As before, we also determined the test error for manipulated cell characteristics, i.e., rotations in CP-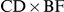 space and CPs set to zero (not shown). In all cases we found the original distribution of CP and CD to clearly provide the best acuity of about 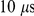. Specifically for the Jeffress-like situation (CP = 0), we find a a-mean-square test error of 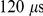, i.e., the maximal ITD.

To conclude, for hemispheric rate difference representation the experimentally observed distribution of CP and 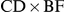 is more suitable in terms of test error and frequency invariance than all artificial ones tested.

## Discussion

Responses of ITD-sensitive neurons in the DNLL of gerbils change with the frequency of a pure tone stimulus, similar to all other ITD sensitive neurons in the brainstem [25]. Here, we have evaluated this frequency-dependent modulation in terms of its influence on the encoding of ITD by firing rate patterns of the neuronal population. For the 153 recorded cells we have characterized the frequency dependence by the two parameters characteristic phase (CP) and characteristic delay (CD) [2]. We found that the two parameters are significantly negatively correlated, as has also been reported for the midbrain and DNLL of guinea pigs [31], [37], although there DNLL data did not reveale negative CDs. Also consistent with these and several other studies in various binaural brainstem nuclei and animals, we found that CPs are broadly distributed over almost the whole phase cycle [3], [6], [28], [38].

Analysis of single-cell mutual information revealed that the observed distribution of CPs and CDs performs slightly better than a distribution with shuffled CPs and CDs. Furthermore, the single-cell mutual information strongly depends on the noise level. We found that for high noise levels peak-based codes are advantageous in terms of mutual information. For low and moderate noise levels we find mixed coding schemes to be viable: Both slopes and peaks can be used to extract information and should be located in the physiological range. These results are consistent with theoretical work comparing slope and peak-based coding schemes [39], [40]. There it is generally shown that for high noise levels strong signal changes are preferred and thus binary-like (i.e., peak-based) codes are beneficial. For low noise, slope-based codes are preferred since only then can continuous rate changes be sampled well enough.

The statistical model allows derivation of hypothetical distributions of CP and CD for different head sizes. As expected, mutual information grows with increasing inter-ear distance. Also the regions of highest mutual information move towards smaller CPs as we increased the inter-ear distance. Interestingly, this effect corresponds well to the finding that for large mammals the medial superior olive (MSO; with most CPs between 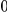 and 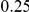 cycles) is generally larger than for smaller mammals [41].

An increase in the inter-ear distance can alternatively be interpreted as an increase of best frequency. In both cases tone delay functions with peaks in the physiological range exhibit increased mutual information. With this interpretation we can also assess the situation when phase-locking is present up to several kHz, as found in the barn owl [4], [42]. There, as well as for a large head diameter, the two regions of high mutual information merge into one cluster centered about CP = CD = 0. As a consequence, a Jeffress-like coding strategy with CP = 0 would be sufficient for achieving high single-cell mutual information.

The variability of phase delay functions in the DNLL provides the basis for a frequency-invariant population representation of ITDs. We find that both of two readout strategies, a linear classifier and a bilateral rate difference signal (two channel code) can explain a coding acuity of down to 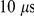. For the linear classifier, however, the observed distribution in CP-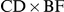 space with BP clustered about 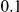 cycles is suboptimal in that a Jeffress-type representation with CP = 0 would account for a better acuity. For the bilateral difference code the observed distribution of CPs and CDs seems appropriate, particularly because of the linearity and the frequency invariance of the difference signal. There, a Jeffress-like representation would yield a much lower acuity.

The behavioral acuity of gerbils at midline (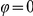) has been estimated as 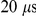 [34], [35] and thus is worse than the acuity of about 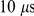 derived from the bilateral difference model. Such hyperacuity of the estimator is not surprising as the relative noise decreases with the size of the population. In general, hyperacuity has two possible explanations. First, it may hint at several noisy downstream readout stations before the localization signal is translated to a behavioral response. As a second possibility, however, it could also hint at hidden stimulus dimensions that are not taken into account by the decoding model. As for the frequency dependence discussed in our paper, one could also ask for a code to be invariant with respect to intensity, background noise etc.. Each of these additional dimensions, hence, reduces the predictive value of single neurons. The real psychophysical acuity should then be achieved by a decoding model that takes into account all possible invariances assuming no further noise in the readout.

Another possible discrepancy to psychophysical data is that the acuity of the bilateral rate difference model is independent of stimulus ITD. In humans the minimal audible angle at lateral (azimuth 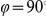) positions is up to 10 times worse than at frontal positions (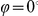) [43]. However, the transformation from angle to ITD only accounts for a factor of about 2 (see Materials and Methods). Indeed, the psychophysical ITD resolution for low-frequency pure tones is about 2 to 5 times worse for lateral positions as for frontal ones [43]. In gerbils, localization acuity has not yet been determined at locations different from midline. However, the bilateral difference model predicts that in gerbils the just noticeable ITD difference is independent of azimuth and conversely the acuity in terms of azimuthal angle should be about 2 times worse for lateral positions than at midline. This feature could be a specialty of animals with small head size, because if the inter-ear distance gets larger more peaks of the phase delay functions move into the physiological range and impair decoding via a difference rate particularly for more lateral positions.

Non-zero CPs are most often thought to originate in the lateral superior olive where neurons receive inhibition from contralateral and excitation from ipsilateral. The combination of these antiphasic signals is able to explain CPs around 0.5 and low CDs. Such cells are generally called troughers. For neurons that receive bilateral excitation (as in the MSO) CPs different from zero still pose a major problem for mechanistic models of ITD sensitivity as the physiological mechanisms that give rise to them are not fully identified, yet. The classical Jeffress model [29] in which the best ITD is solely determined by temporal latency differences predicts constant CP = 0. Cells with small CPs are generally called peakers. There are several candidate models for non-zero CPs in binaurally excited neurons. 1) Ipsi- and contralateral input fibers might have mismatched center frequencies and thus a mismatch of phases might be induced by the preprocessing of different cochlear filters [44]. 2) Morphological asymmetries [45] of the coincidence detecting neuron can induce distinct temporal filtering of the ipsi- and contralateral inputs. 3) Phase-locked inhibition [7], [26], [46], [47] that differs between ipsi- and contralateral input can induce asymmetric phase shifts. 4) Phase disparities may be a direct consequence of asymmetries in the ipsi- and contralateral excitatory synaptic kinetics [48]. The present study shows that generating specific CPs may not just be an epiphenomenon of the physiological mechanisms that underlie ITD-sensitive responses in the brainstem but may be required for an optimal neuronal representation of ITD. Thus the physiological mechanisms underlying ITD sensitivity should allow the deliberate tuning of CPs, which argues against hard-wired solutions as (1) and (2) and favors synaptic mechanisms like (3) and (4).

A problem in the interpretation of our data is that the DNLL is not a primary nucleus in which the ITD-sensitive responses are computed. The ITD representation in the DNLL might already be imposed by secondary processing steps. Instead one would rather want to compare population responses in the MSO (for low CPs) and the low-frequency region of the lateral superior olive (for high CPs). Single units in the MSO are, however, difficult to record from. Data from a few tens of gerbil MSO units also shows negatively correlated CP and CD with a broad distribution of CPs (unpublished observation about data from [27]). The DNLL, however, is a particularly good place to study ITD population codes, since it is much easier to record from than the MSO and, moreover, it is the first station in which genuine ITD-sensitive responses from MSO (peakers) and lateral superior olive (troughers) are combined [25]. The only major computation occurring at the synapses from the superior olivary complex to the DNLL seems to be noise reduction [21].

Most theoretical analyzes of neuronal representations deal with only one or two stimulus dimensions as e.g. the frequency of a tone or the loudness of a sound. In the example discussed in the present paper the two stimulus dimensions ITD and frequency are both physically and statistically independent since sound position and sound spectrum are generally unrelated. Here, we have shown that considering population responses across an invariant dimension (frequency) of the stimulus not only allows the assessment of the neuronal population representation in terms of coding acuity, but also allows to evaluate, how different hypothetical invariant read-out strategies fit to the population representation.

## Materials and Methods

### Ethics statement

All experiments were approved according to the German Tierschutzgesetz (AZ 55.2-1-54-2531-57-05).

### Animals and recordings

Single neurons (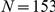) in the DNLL were recorded from 41 Mongolian gerbils (Meriones unguiculatus) of both sexes 2–3 months of age. The data have already been used for previous publications [24], [25]. There, detailed methods in terms of surgical preparation, acoustic stimulus delivery, stimulus calibration, and recording techniques have been described.

### Stimuli

Stimuli were generated at 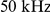 sampling rate by TDT System II or III (Tucker Davis Technologies). Digitally generated stimuli were converted to analog signals (DA3-2/RP2-1, TDT), attenuated (PA5, TDT), and delivered to the ear phones (Sony, Stereo Dynamic Earphones, MDR-EX70LP).

The standard setting was stimulus duration of 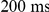 plus cosine rise/fall times of 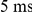, presented at a repetition rate of 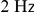. To search for acoustically evoked responses, binaurally uncorrelated noise stimuli were delivered. When a neuron was encountered, first its best frequency (BF) and absolute threshold was determined using binaurally identical pure tone stimulation. The frequency that elicited responses at the lowest sound intensity was defined as BF, the lowest sound intensity evoking a noticeable response at BF as threshold. Sensitivity to interaural time differences (ITDs) was primarily assessed by presenting a matrix of pure-tone stimuli with varying ITDs and stimulus frequencies 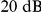 above threshold. Different ITDs were presented over a range equivalent to at least one cycle of the stimulus frequency 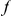. ITD sensitivity was tested for 5 frequencies around BF (covering 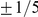 of an octave) and an interaural intensity difference of 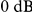. Each stimulus was repeated at least three times.

### Tone delay functions

Tone delay functions describe the firing rate of a neuron as a function of the stimulus ITD for a fixed stimulus frequency 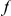. In this paper, for the purpose of a simpler notation, we consider tone delay functions to depend on the interaural phase difference (IPD) 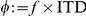. The rates were averaged over all repetitions of the respective pure tone stimulus and fitted by the cyclic Gaussian
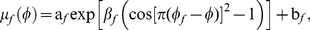
(3)providing four fit parameters 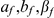, and 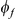. The parameter 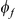 accounts for the IPD at which the fit has its maximum value and is called the best IPD 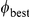. Note that also negative values for the best phase can occur and are kept as such in our analysis.

### Circular-linear regression

The best IPD 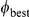 as a function of the frequency 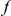 of the pure tone stimulus is called phase-frequency curve. It relates a circular (phase) variable, 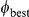 to a linear variable 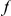. This relation is used to derive single cell characteristic phase (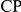) and characteristic delay (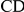) using Equation (1). Quantification of correlations between CP and CD in the population of cells (Figure 1E) also requires to assess the relation between a circular variable (CP) and a linear variable (CD).

To fit linear relations between pairs of measurements 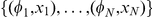, in which the dependent variable 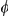 is a circular quantity (e.g., CP, or IPD), and the independent variable 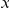 is linear (e.g., CD, or frequency), we follow the approach by Schmidt et al. (2009) [49]: Assuming the linear model 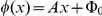, one computes the mean resultant length 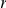 of the circular errors between the measurements 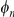 and the model 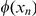:
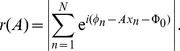
(4)


If the model exactly fitted the data 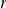 would take the maximal value 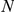. Since in Equation (4) the dependence on the phase offset parameter 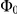 cancels out, the slope parameter 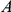 can be obtained from one-dimensional numerical maximization of 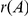. For the resulting optimal slope 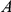, the offset 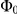 then follows from maximizing
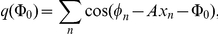
which accounts for maximizing the overlap between the data cloud and the linear fit on the surface of a cylinder. Maximization of 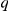 was already suggested by Agapiou and Mc Alpine (2008) [31] for fitting CP. Significance and correlation coefficients of circular linear fits was evaluated using the Matlab package circstat [30].

### Prior distribution

To obtain the mutual information between stimulus position and single cell firing rate according to equation (2), we require a model for the probability distribution 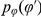 of the interaural angles of the sound sources.

A uniform distribution of the dihedral angles of the sound sources corresponds to a distribution 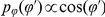 of interaural angles 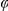 on the great circle defined by the sound source elevation. For zero elevation, 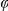 is equivalent to the azimuth. Following Blauert [12], the interaural angle is mapped to the ITD 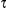 via
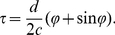
(5)


Unless otherwise mentioned, we use an inter-ear distance of 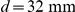 for the gerbil. Together with the speed of airborne sound 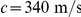 this leads to a physiological range of ITDs of 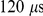. The prior distribution of ITDs is then obtained as
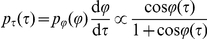
in which 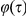 is the numerical inverse of equation (5).

### Rate distributions

To obtain the mutual information between firing rate and stimulus position following the procedure described after equation (2), we require an estimate for the conditional probability distribution 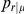 of observing a firing rate 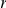 given a stimulus that evoked mean response rate 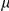. The corresponding rate histograms were constructed cell-wise for each mean firing rate 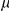 and fitted by a Gaussian (Figure 2B),
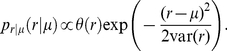
(6)


Here the step function 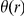 is included to clip negative firing rates, it equals 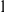 for 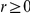 and zero for 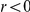. The variance 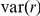 (pooled over over all cells) could be fitted by a logarithmic relation
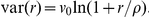



For a stimulus length of 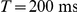 the fit parameters were 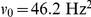 and 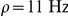. For small rates the logarithm can be expanded and leads to the approximate relation 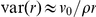. The variance of the spike count 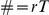 thus becomes 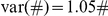, which is Poissonian to an excellent approximation. For large rates the variance of the spike count increases strongly sublinear meaning that DNLL cells encode much more faithfully than Poisson at high rates [21].

### Linear classifiers

To evaluate the possibility of a population representation of ITDs via grandmother neurons, we defined 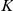 categories (labels) which correspond to ITDs being in intervals of size 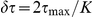. Grandmother neurons are assumed to respond to ITDs from only one of these bins. Using the machine learning package Shogun [33], we learned the weights 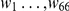 of linear decision variables
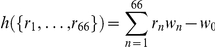
with input data 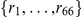 generated by the stochastic model that was fitted to the DNLL rate responses. Training was done in a one-vs.-one mode, i.e., for each pair of bins we trained a support vector machine (SVM) to distinguish between those two categories. Thus, for 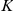 ITD bins a total of 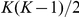 SVMs had to be trained. The estimated ITD bin 
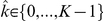
 of the pattern is the one which has the most votes from the 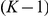 SVMs that were trained to classify it. The grandmother neurons are thus assumed to implement a winner-take-all based on the number of votes. The (root mean square) test error on a set of 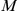 test inputs is computed as
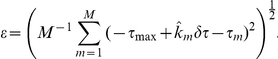



## Supporting Information

Figure S1Robustness of single cell mutual information. (A–D) Single cell mutual information for different best frequencies. White circles represent measured CP, 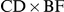 values for the cells in the respective best frequency band. (E,F) Single cell mutual information for uniform (E) and power-law (F) frequency distribution (E is the same grey level plot as C).(PDF)

Figure S2Robustness of linear rate difference signal. Left column: Linear rate difference code for a model population at which the CP, 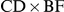 values are jittered around the measured values according to a Gaussian distributions with standard deviation 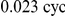. for CP and 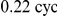. for 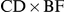 (see Supporting Information Figure S3). Right column: Linear rate difference code for the 41 cells in the (best) frequency band between 600 and 800 Hz. The arrangement of the sub panels is identical to those in Figure 5 of the main paper.(PDF)

Figure S3Robustness of CP, CD estimate. (A) Measured distribution (white) and one example of a surrogate distribution (black) obtained by randomly generating spike counts from a Gaussian distribution with cell-specific mean and variance. (B,C) Four surrogate spike counts (like in A) were generated for each cell and used to derive cell-wise standard errors of the mean (SEM) for CP and 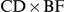. (B) Cumulative distribution of SEM for CP (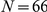 cells). Vertical lines indicate the SEM values of about 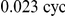. and 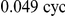. at the 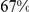 and 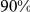 quantile, respectively. (C) Same as B for 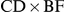, with SEM values 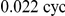. and 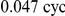. at the 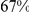 and 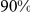 quantiles.(PDF)

## References

[1] Goldberg JM, Brown PB (1969). Response of binaural neurons of dog superior olivary complex to dichotic tonal stimuli: some physiological mechanisms of sound localization.. J Neurophysiol.

[2] Yin TC, Kuwada S (1983). Binaural interaction in low-frequency neurons in inferior colliculus of the cat. III. Effects of changing frequency.. J Neurophysiol.

[3] Yin TC, Chan JC (1990). Interaural time sensitivity in medial superior olive of cat.. J Neurophysiol.

[4] Carr CE, Konishi MA (1990). Circuit for detection of interaural time differences in the brainstem of the barn owl.. J Neurosci.

[5] McAlpine D, Jiang D, Palmer AR (2001). A neural code for low-frequency sound localization in mammals.. Nat Neurosci.

[6] Fitzpatrick DC, Kuwada S (2001). Tuning to interaural time differences across frequency.. J Neurosci.

[7] Brand A, Behrend O, Marquardt T, McAlpine D, Grothe B (2002). Precise inhibition is essential for microsecond interaural time difference coding.. Nature.

[8] Köppl C, Carr CE (2006). Maps of interaural time difference in the chicken's brainstem nucleus laminaris.. Biol Cybern.

[9] Carr CE, Soares D, Smolders J, Simon JZ (2009). Detection of interaural time differences in the alligator.. J Neurosci.

[10] Rayleigh LJ (1907). On our perception of sound direction.. Philos Mag.

[11] Wightman FL, Kistler DJ (1992). The dominant role of low-frequency interaural time differences in sound localization.. J Acoust Soc Am.

[12] Blauert J (1997). Spatial hearing: The psychophysics of human sound localization..

[13] Tollin D, Yin TC (2005). Interaural phase and level difference sensitivity in low-frequency neurons in the lateral superior olive.. J Neurosci.

[14] Carr CE, Soares D, Parameshwaran S, Perney T (2001). Evolution and development of time coding systems. Curr Opin Neurobiol..

[15] Grothe B (2003). New roles for synaptic inhibition in sound localization.. Nat Rev Neurosci.

[16] Köppl C (2009). Evolution of sound localisation in land vertebrates.. Curr Biol.

[17] McAlpine D, Grothe B (2003). Sound localization and delay lines – do mammals fit the model?. Trends in Neurosci.

[18] Harper NS, McAlpine D (2004). Optimal neural population coding of an auditory spatial cue.. Nature.

[19] Shackleton TM, Skottun BC, Arnott RH, Palmer AR (2003). Interaural time difference discrimination thresholds for single neurons in the inferior colliculus of Guinea pigs.. J Neurosci.

[20] Skottun BC, Shackleton TM, Arnott RH, Palmer AR (2001). The ability of inferior colliculus neurons to signal differences in interaural delay.. Proc Natl Acad Sci USA.

[21] Pecka M, Siveke I, Grothe B, Lesica NA (2009). Enhancement of ITD coding within the initial stages of the auditory pathway Enhancement of ITD coding in the auditory pathway.. J Neurophysiol.

[22] Palmer AR, Liu LF, Shackleton TM (2007). Changes in interaural time sensitivity with interaural level differences in the inferior colliculus.. Hear Res.

[23] Palmer AR, Jiang D, McAlpine D (1999). Desynchronizing responses to correlated noise: a mechanism for binaural masking level differences at the inferior colliculus.. J Neurophysiol.

[24] Siveke I, Leibold C, Grothe B (2007). Spectral composition of concurrent noise affects neuronal sensitivity to interaural time differences of tones in the dorsal nucleus of the lateral lemniscus.. J Neurophysiol.

[25] Siveke I, Pecka M, Seidl AH, Baudoux S, Grothe B (2006). Binaural response properties of low-frequency neurons in the gerbil dorsal nucleus of the lateral lemniscus.. J Neurophysiol.

[26] Batra R, Kuwada S, Fitzpatrick DC (1997). Sensitivity to interaural temporal disparities of low- and high-frequency neurons in the superior olivary complex. I. Heterogeneity of responses.. J Neurophysiol.

[27] Pecka M, Brand A, Behrend O, Grothe B (2008). Interaural time difference processing in the mammalian medial superior olive: the role of glycinergic inhibition.. J Neurosci.

[28] Kuwada S, Fitzpatrick DC, Batra R, Ostapoff E-M (2006). Sensitivity to interaural time differences in the dorsal nucleus of the lateral lemniscus of the unanesthetized rabbit: Comparison with other structures.. J Neurophysiol.

[29] Jeffress LA (1948). A place theory of sound localization.. J Comp Physiol.

[30] Berens P (2009). CircStat: A MATLAB toolbox for circular statistics.. J Stat Software.

[31] Agapiou JP, McAlpine D (2008). Low-frequency envelope sensitivity produces asymmetric binaural tuning curves.. J Neurophysiol.

[32] Vapnik V (1998). Statistical learning theory..

[33] Sonnenburg S, Raetsch G, Schaefer C, Schoelkopf B (2006). Large scale multiple kernel learning.. J Mach Learn Res.

[34] Heffner RS, Heffner HE (1988). Sound localization and use of binaural cues by the gerbil (Meriones unguiculatus).. Behav Neurosci.

[35] Lesica N, Lingner A, Grothe B (2010). Population coding of interaural time differences in gerbils and barn owls.. J Neurosci.

[36] Marquardt T, McAlpine D, Kollmeier B, Klump G, Hohmann V, Langemann U, Mauermann M, Uppenkamp S, Verhey J (2006). A *π*-limit for coding ITDs: Implications for binaural models.. Hearing From Sensory Processing to Perception.

[37] McAlpine D, Jiang D, Palmer AR (1996). Interaural delay sensitivity and the classification of low best-frequency binaural responses in the inferior colliculus of the guinea pig.. Hear Res.

[38] Batra R, Fitzpatrick DC (2002). Processing of interaural temporal disparities in the medial division of the ventral nucleus of the lateral lemniscus.. J Neurophysiol.

[39] Bethge M, Rotermund D, Pawelzik K (2003). Second order phase transition in neural rate coding: binary encoding is optimal for rapid signal transmission.. Phys Rev Lett.

[40] Butts DA, Goldman MS (2006). Tuning curves, neuronal variability, and sensory coding.. PLoS Biol.

[41] Glendenning KK, Masterton RB (1998). Comparative morphometry of mammalian central auditory systems: variation in nuclei and form of the ascending system.. Brain Behav Evol.

[42] Köppl C (1997). Phase locking to high frequencies in the auditory nerve and cochlear nucleus magnocellularis of the barn owl, Tyto alba.. J Neurosci.

[43] Mills AW (1958). On the minimum audible angle.. J Acoust Soc Am.

[44] Joris PX, Van de Sande B, Louage DH, van der Hejden M (2006). Binaural and cochlear disparities.. Proc Natl Acad Sci USA.

[45] Zhou Y, Carney LH, Colburn HS (2005). A model for interaural time difference sensitivity in the medial superior olive: interaction of excitatory and inhibitory synaptic inputs, channel dynamics, and cellular morphology.. J Neurosci.

[46] Leibold C, van Hemmen JL (2005). Spiking neurons learning phase delays: how mammals may develop auditory time-difference sensitivity.. Phys Rev Lett.

[47] Leibold C (2010). Influence of inhibitory synaptic kinetics on the interaural time difference sensitivity in a linear model of binaural coincidence detection.. J Acoust Soc Am.

[48] Jercog PE, Svirskis G, Kotak VC, Sanes DH, Rinzel J (2010). Asymmetric excitatory synaptic dynamics underlie interaural time difference processing in the auditory system.. PLoS Biol.

[49] Schmidt R, Diba K, Leibold C, Schmitz D, Buzsaki G (2009). Single-trial phase precession in the hippocampus.. J Neurosci.

