# Supplementary material for: Frequency-Invariant Representation of Interaural Time Differences in Mammals
Source: PLoS Comput Biol. 2011 Mar 17;7(3):e1002013. doi: 10.1371/journal.pcbi.1002013 (PMC3060160; doi:10.1371/journal.pcbi.1002013)
Supplement: Figure S1 — Robustness of single cell mutual information. (A–D) Single cell mutual information for different best frequencies. White circles represent measured CP, values for the cells in the respective best frequency band. (E,F) Single cell mutual information for uniform (E) and power-law (F) frequency distribution (E is the same grey level plot as C). (PDF) [file pcbi.1002013.s001.pdf]

Figure S1: Robustness of single cell mutual information

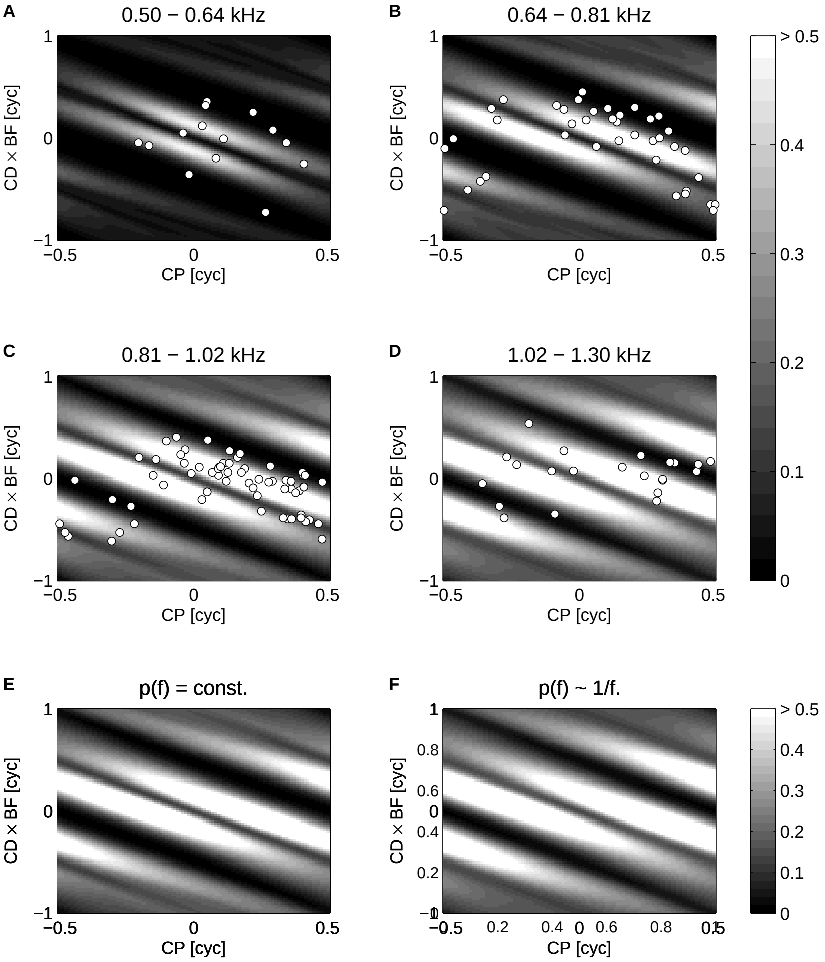

Figure S1: (A-D) Single cell mutual information for different best frequencies. White circles represent measured CP, CD×BF values for the cells in the respective best frequency band. (E,F) Single cell mutual information for uniform (E) and power-law (F) frequency distribution (E is the same grey level plot as C).
