# Supplementary material for: Frequency-Invariant Representation of Interaural Time Differences in Mammals
Source: PLoS Comput Biol. 2011 Mar 17;7(3):e1002013. doi: 10.1371/journal.pcbi.1002013 (PMC3060160; doi:10.1371/journal.pcbi.1002013)
Supplement: Figure S2 — Robustness of linear rate difference signal. Left column: Linear rate difference code for a model population at which the CP, values are jittered around the measured values according to a Gaussian distributions with standard deviation . for CP and . for (see Supporting Information Figure S3). Right column: Linear rate difference code for the 41 cells in the (best) frequency band between 600 and 800 Hz. The arrangement of the sub panels is identical to those in Figure 5 of the main paper. (PDF) [file pcbi.1002013.s002.pdf]

Figure S2: Robustness of linear rate difference signal

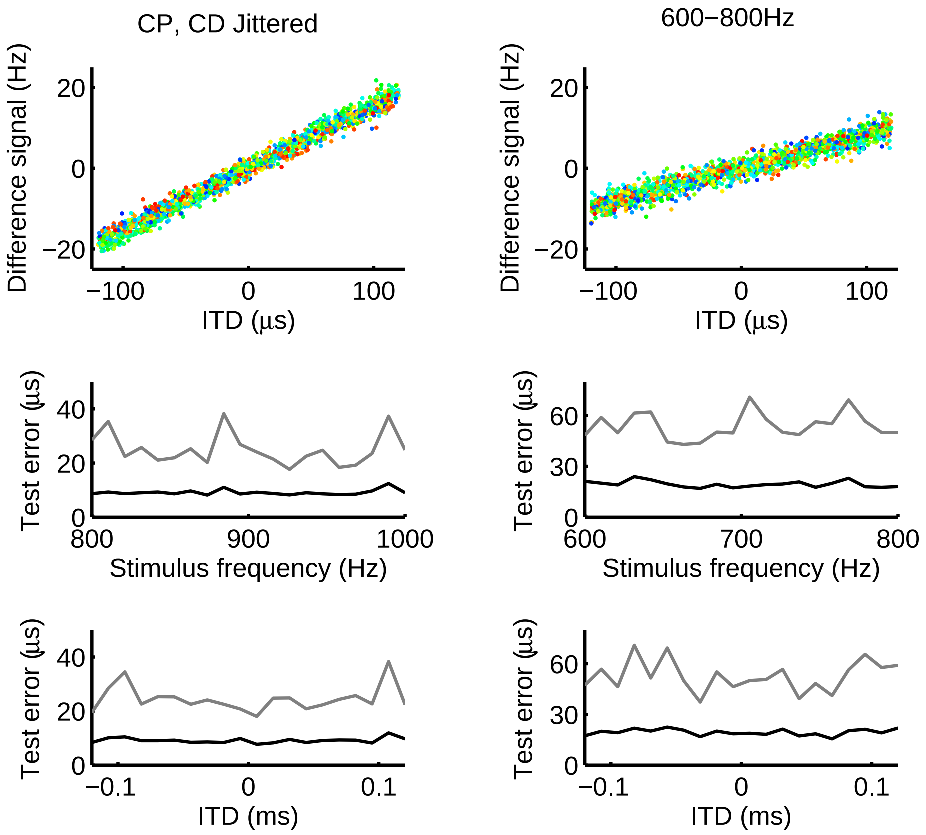

Figure S2: Left column: Linear rate difference code for a model population at which the CP, CD×BF values are jittered around the measured values according to a Gaussian distributions with standard deviation 0.023 cyc. for CP and 0.22 cyc. for CD×BF (see Supporting Information Figure S3). Right column: Linear rate difference code for the 41 cells in the (best) frequency band between 600 and 800Hz. The arrangement of the sub panels is identical to those in Figure 5 of the main paper.
