# Supplementary material for: Frequency-Invariant Representation of Interaural Time Differences in Mammals
Source: PLoS Comput Biol. 2011 Mar 17;7(3):e1002013. doi: 10.1371/journal.pcbi.1002013 (PMC3060160; doi:10.1371/journal.pcbi.1002013)
Supplement: Figure S3 — Robustness of CP, CD estimate. (A) Measured distribution (white) and one example of a surrogate distribution (black) obtained by randomly generating spike counts from a Gaussian distribution with cell-specific mean and variance. (B,C) Four surrogate spike counts (like in A) were generated for each cell and used to derive cell-wise standard errors of the mean (SEM) for CP and . (B) Cumulative distribution of SEM for CP ( cells). Vertical lines indicate the SEM values of about . and . at the and quantile, respectively. (C) Same as B for , with SEM values . and . at the and quantiles. (PDF) [file pcbi.1002013.s003.pdf]

Figure S3: Robustness of CP CD estimate

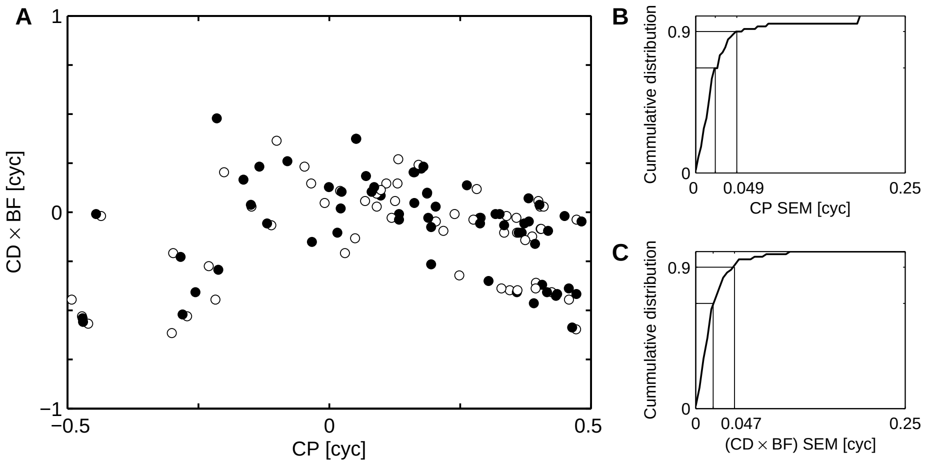

Figure S3: (A) Measured distribution (white) and one example of a surrogate distribution (black) obtained by randomly generating spike counts from a Gaussian distribution with cell-specific mean and variance. (B,C) Four surrogate spike counts (like in A) were generated for each cell and used to derive cell-wise standard errors of the mean (SEM) for CP and CD×BF. (B) Cumulative distribution of SEM for CP (N = 66 cells). Vertical lines indicate the SEM values of about 0.023 cyc. and 0.049 cyc. at the 67% and 90% quantile, respectively. (C) Same as B for CD×BF, with SEM values 0.022 cyc. and 0.047 cyc. at the 67% and 90% quantiles.
